# Patterning the Stiffness of Elastomeric Nanocomposites by Magnetophoretic Control of Cross-linking Impeder Distribution

**DOI:** 10.3390/ma8020474

**Published:** 2015-01-30

**Authors:** Suvojit Ghosh, Mehran Tehrani, Marwan S. Al-Haik, Ishwar K. Puri

**Affiliations:** 1 Department of Engineering Physics, McMaster University, 1280 Main Street West, Hamilton, ON L8S 4L7, Canada; E-Mail: ikpuri@mcmaster.ca; 2 Department of Engineering Science and Mechanics, Virginia Tech, 495 Old Turner Street, Blacksburg, VA 24061, USA; E-Mail: alhaik@vt.edu; 3 Department of Mechanical Engineering, University of New Mexico, Albuquerque, NM 87131, USA; E-Mail: mtehrani@unm.edu; 4 Department of Mechanical Engineering, McMaster University, 1280 Main Street West, Hamilton, ON L8S 4L7, Canada

**Keywords:** functional grading, nano composites, nanoindentation, magnetic nanoparticles

## Abstract

We report a novel method to pattern the stiffness of an elastomeric nanocomposite by selectively impeding the cross-linking reactions at desired locations while curing. This is accomplished by using a magnetic field to enforce a desired concentration distribution of colloidal magnetite nanoparticles (MNPs) in the liquid precursor of polydimethysiloxane (PDMS) elastomer. MNPs impede the cross-linking of PDMS; when they are dispersed in liquid PDMS, the cured elastomer exhibits lower stiffness in portions containing a higher nanoparticle concentration. Consequently, a desired stiffness pattern is produced by selecting the required magnetic field distribution *a priori*. Up to 200% variation in the reduced modulus is observed over a 2 mm length, and gradients of up to 12.6 MPa·mm^−1^ are obtained. This is a significant improvement over conventional nanocomposite systems where only small unidirectional variations can be achieved by varying nanoparticle concentration. The method has promising prospects in additive manufacturing; it can be integrated with existing systems thereby adding the capability to produce microscale heterogeneities in mechanical properties.

## 1. Introduction

Heterogeneities in mechanical properties, also known as functional grading, enhances the mechanical performance of materials [1]. Complex heterogeneities with nano- and micro-meter length scales are of particular significance in structural materials, e.g., they enhance damage tolerance in tough biomaterials such as bones [2,3]. Existing methods to produce heterogeneities, however, are archaic as they only offer unidirectional variations [4]. Here, we present a novel method to enforce any arbitrary pattern in the stiffness of an elastomeric material. This is achieved by selectively impeding cross-linking reactions at desired locations while curing. High spatial resolutions with sharp gradients can be achieved; we demonstrate up to 200% variation in the stiffness over a 2 mm length.

Wide adoption of polymeric materials has motivated several methods for functional grading in polymer composites. Such a composite typically comprises a filler phase (A) distributed in a polymeric matrix (B). Gradients in A:B proportions induce spatial variations in material properties [5]. Grading is often achieved only with discrete steps which limit spatial resolution, e.g., during compression molding of multiple sheets containing composition variations [6], or by layer-wise curing of co-polymer blends in varying proportions [7]. Continuous grading can be accomplished only when A and B have different densities, e.g., employing centrifugal or gravity forces to induce variations in the A:B ratio [8,9,10,11]. At smaller length scales, continuous grading can be enforced with diffusion which allows limited control [12,13]. Other methods such as melt-mixing of co-polymers [14] and sophisticated extrusion processes [15] have also been explored. However, none of these allow patterning beyond a unidirectional gradient. Herein, we overcome this limitation by using a magnetic field to enforce concentration variations.

Use of magnetic nanoparticles is popular because they can be remotely manipulated using a magnetic field [16,17,18,19,20,21,22]. For instance, it finds application in targeted drug delivery wherein the nanoparticles are conjugated with bio-molecules [23,24]. Control over spatial distribution of such nanoparticle-conjugates enables localized reactions [25,26]. We present a method which allows such spatial control over reactions with the intent to pattern stiffness. Cross-linking of polydimethylsiloxane (PDMS) occurs through the hydrosilylation reaction which is catalyzed, for example, by the platinum present in Karstedt’s catalyst [27,28,29]. When colloidal magnetite nanoparticles (MNPs) are dispersed in liquid PDMS, they impede the cross-linking reactions [30]. This degrades the stiffness of the resulting elastomer [31,32]. We use an external magnetic field to enforce a desired concentration distribution of MNPs in liquid PDMS while curing. This enables us to enforce a desired pattern of stiffness in PDMS.

MNP-polymer nanocomposites are synthesized by solution-casting the polymers after dissolving them in a ferrofluid, *i.e.*, a colloidal dispersion of MNPs [33,34]. An external magnetic field can be used to organize the MNPs into a desired microstructure in the solution [35]. The solvent is subsequently evaporated and the polymer is cured under exposure to a magnetic field, thus preserving the microstructure. This enables a ‘material printer’; beginning with the same polymer-MNP solution, an external magnetic field is used to vary the material properties, resulting in different manifestations of the final material [36,37,38,39]. However, previous studies have been restricted to the control of bulk mechanical [37], magnetic [36] and electrical [40] properties of a material.

**Figure 1:**
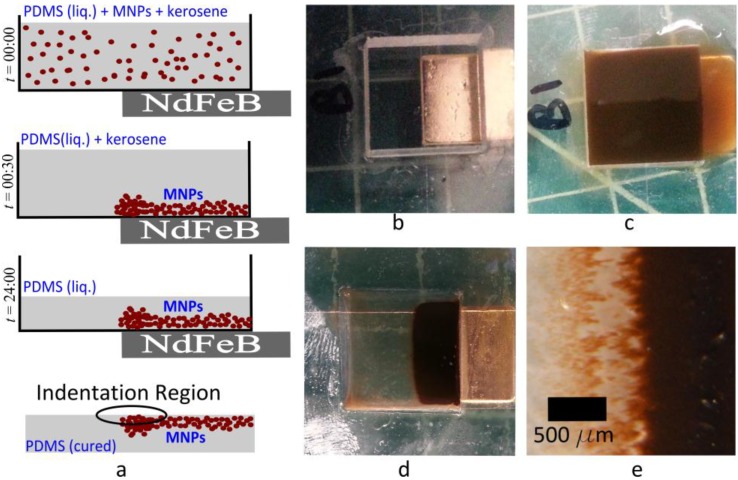
Control of magnetite nanoparticle (MNP) concentration distribution to pattern stiffness of polydimethysiloxane (PDMS). (**a**) PDMS is dissolved in a kerosene-based ferrofluid containing MNPs (shown disproportionately large). The solution is cast into a mold adjacent to a permanent magnet. Within ~30 min, all MNPs move to the region adjacent to the magnet. The kerosene is then evaporated and the polymer cured, thus preserving the MNP concentration distribution. Instrumented nanoindentation is performed in the region with the highest gradient of MNP concentration; (**b**) Acrylic mold with a coverslip as its floor, showing an NdFeB magnet covering part of the floor; (**c**) Solution of PDMS in kerosene is injected into the mold; (**d**) After ~30 min, MNPs concentrate in the region above the magnet; (**e**) Magnified image of the boundary, showing a sharp gradient in MNP concentration.

We earlier reported a solution casting method for easy control of the concentration distribution of MNPs in PDMS [35]. PDMS was first dissolved in a ferrofluid, the solvent was then evaporated and the polymer cured while being exposed to a magnetic field. Spatial variations in field strength facilitated a desired concentration distribution of MNPs in the finished elastomer. However, initially the entire PDMS had a homogeneous nanoparticle concentration. Thus, it was uncertain if the gradients in MNP concentration that were eventually produced enabled selective impeding of cross-linking at desired locations in the PDMS elastomer and thereby produced sharp gradients in the stiffness of the solid. Here, using instrumented nanoindentation, we demonstrate the capability to produce high resolution patterns of stiffness. The method is illustrated in Figure 1.

## 2. Results and Discussion

Cross-linking in PDMS occurs through the hydrosilylation reaction where linear chains of PDMS react with a cross-linker yielding a 3D elastomer matrix [29,41]. This reaction is catalyzed, e.g., by Karsted’s catalyst, an organometallic complex containing platinum [27,28]. The cross-linking reaction is sensitive to additives in liquid PDMS. Particularly, addition of MNPs are known to impede cross-linking [30]. The exact mechanism of impedance is not known but various factors have been suggested, e.g., pollution of Karsted’s catalyst by the MNPs [31] or the complex interactions between the MNP surface and the polymer chains [32,42]. When MNPs are dispersed in liquid PDMS, the bulk elastic modulus *E* of the cured elastomer is degraded [31,32] possibly due to the resulting decrease in cross-link density [29]. However, there are insufficient data to clearly demonstrate the trends in the variation of *E* with MNP mass fraction (*ψ*), particularly within the range of *ψ* that we have used. Our materials and methods also differ significantly from previous reports. Thus, our first step was to determine the influence of MNP addition on the stiffness of PDMS.

**Figure 2:**
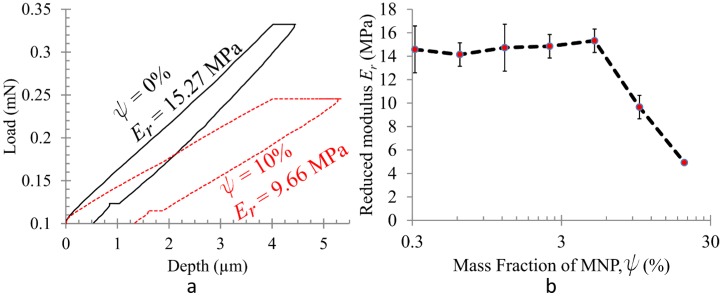
Effect of magnetite nanoparticle addition on the reduced modulus (*E*_r_), determined from homogeneous composite samples. (**a**) Pure PDMS is much stiffer than PDMS cured with *ψ =* 10% (*w*/*w*) MNPs, as determined from the slope of force-displacement curves obtained by instrumented nanoindentation; (**b**) *E*_r_ does not change appreciably with *ψ* up to *ψ* = 5%. For *ψ* > 5%, impeding of crosslinking becomes noticeable and *E*_r_ decreases monotonically with further increase of *ψ*.

To determine the efficacy of MNPs in degrading stiffness, we prepared composite samples that were cured in the absence of a magnetic field, *i.e.*, these are expected to contain a relatively homogeneous distribution of MNPs [37,39,43]. Representative indentation data and the reduced modulus (*E*_r_) calculated from the same are presented in Figure 2. There is no appreciable variation in *E*_r_ with *ψ* up to *ψ* = 5% although the data may indicate a slight increase. However, at higher MNP concentrations, the cross-link impeding effect of MNPs dominate. This possibly reduces the cross-link density produced in the finished elastomer [30]. Thus, *E*_r_ decreases monotonically with *ψ* thereafter. For *ψ* >20%, the impedance to cross-linking reaches a level where curing is no longer possible, *i.e.*, the sample remains a liquid. This observation is consistent with earlier reports [31].

We note a common feature of nanocomposites which contain fillers that are significantly stiffer than the polymer matrix. These materials exhibit a rapid rise in their elastic modulus as the particle concentration crosses the percolation threshold. This behavior occurs because the nanoparticles form a continuous network throughout the entire material due to aggregation [40,44]. However, the jump in elastic modulus is often of an order of magnitude or more [45,46]. We did not observe this feature for the range of *ψ* used in our investigation (Figure 2b). Thus, although some particle aggregation and clustering is observed, the effect of percolation can be ignored.

**Figure 3:**
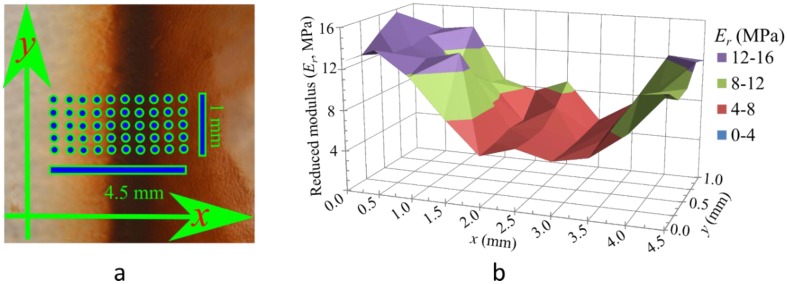
Heterogeneity in *E*_r_. (**a**) Sample with ψ* = 0.1% shows that MNP concentration is highest in the region immediately above the periphery of the magnet. Approximate locations of indentations are shown with dots; (**b**) Map of *E*_r_ obtained from indentation at such points on a sample with ψ* = 1.0% shows that variations in *E*_r_ roughly match variations in *ψ*.

Next, we determined if heterogeneities in MNP concentration produced by the gradual magnetophoretic transport, lasting up to 30 min, was sufficient to produce heterogeneities in the stiffness of the finished material. In order to do so, we used molds with attached permanent magnets, as shown in Figure 1. The resulting heterogeneity in *E*_r_ was determined by performing indentations over an array of points on the region of the sample with the largest gradient in MNP concentration, *i.e.*, on the sample surface that was closest to the edge of the magnet while curing. The results are presented in Figure 3. To cover a large area, we first used a coarse grid with Δ*x =* 500 µm and Δ*y =* 250 µm spanning 4.5 mm in the *x*-direction and 1 mm in the *y*-direction. We denote the mass fraction of MNPs relative to PDMS in the starting solution by ψ*. Heterogeneities in *ψ* are patterned using three different values of the starting concentration, *i.e.*, ψ* = 0.1%, 0.5% and 1.0%. The MNP distribution is visibly most clear in the sample with ψ* = 0.1%, presented in Figure 3a. A band of MNPs is observed in the sample obtained from the region immediately above the magnet’s edge. Approximate positions of the indentations are illustrated on this image. Figure 3b shows that variations in *E*_r_ roughly follow the concentration distribution of MNPs. The material is most stiff, *i.e.*, *E*_r_ is highest in the region that contains pure PDMS (*ψ* = 0). *E*_r_ decreases gradually along the *x-*direction due to the increase in *ψ*. The stiffness again rises beyond the band of high *ψ*. The starting concentration ψ* = 1.0% produces the most distinct heterogeneities. A higher ψ* results in a band of uncured PDMS above the magnet’s edge because the local MNP concentration therein possibly exceeds the *ψ* = 20% threshold described earlier.

**Figure 4:**
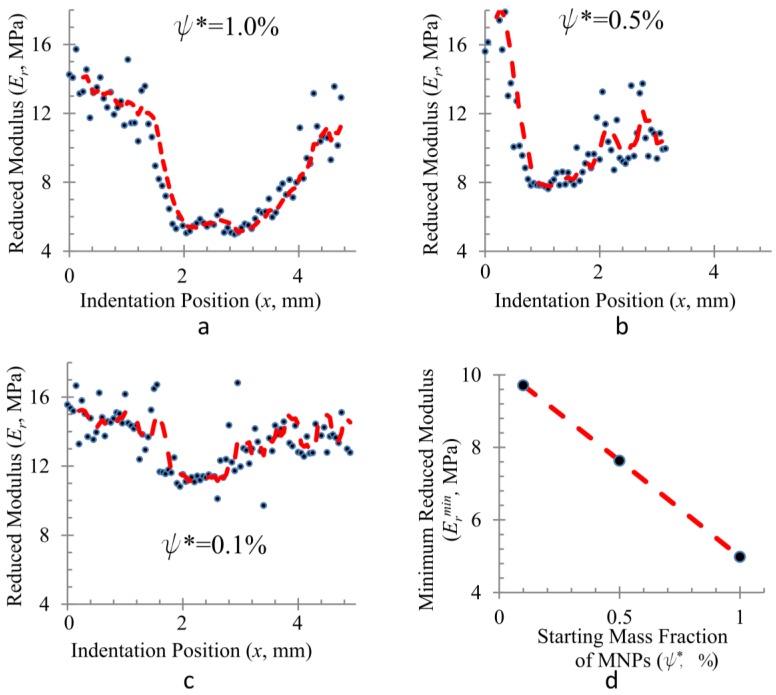
High resolution scan of reduced modulus along a straight line for samples with (**a**) ψ* = 1.0%; (**b**) ψ* = 0.5%; (**c**) ψ* = 0.1% shows that high gradients of the reduced modulus can be formed. The steepest gradient in (**a**) is 12.6 MPa·mm^−1^ (*R*^2^ = 0.9623). The general U-shaped profile is seen in all samples. The red dashed line is a 5-point moving average; (**d**) The minimum reduced modulus (*E*_r_^min^) decreases linearly with increasing ψ*.

Subsequently, in order to determine the resolution of the method, we measured *E*_r_ with finer spatial resolution. Such measurements were obtained from indentations made along a straight line normal to the band of high MNP concentration with Δ*x =* 50 µm. As presented in Figure 4, the highest gradient in *E*_r_ occurs for ψ* = 1.0% where *E*_r_ ranges from ~5 MPa to ~15 MPa within a span of 2 mm. The steepest gradient, 12.6 MPa·mm^−1^, is obtained by fitting a straight line over 10 consecutive data points (*R*^2^ = 0.96). The gradient decreases for lower values of ψ*. While the trend is clear, significant noise in the data prevents an accurate enumeration of any metrics for the gradient. In homogeneous samples, the noise is eliminated by repeating the indentation multiple times at various locations. However, in a heterogeneous sample, this is not possible. The general “U” shape in the *E*_r_ profile is observed for all three values of ψ*. The minimum reduced modulus *E*_r_^min^, which is the lowest for the highest value of ψ*, decreases linearly with increasing ψ* as seen in Figure 4d. The width of U also increases with increasing ψ*.

## 3. Experimental Section

### 3.1. Synthesis of Magnetic Nanoparticles and Ferrofluid

MNPs are synthesized by coprecipitation of Fe^2+^ and Fe^3+^ chlorides in an alkaline solution [47,48]. 44.5 g FeCl_2_·4H_2_O (ACS Grade, A16327, Alfa Aesar, Ward Hill, MA, USA) and 120.8 g FeCl_3_·6H_2_O (ACS Grade, 12497, Alfa Aesar) are dissolved in 300 mL of degassed deionized water (LabChem, ASTM Type II, Zelienople, PA, USA). The solution is added slowly to 372 mL NH_4_OH with vigorous stirring. A black precipitate of MNPs is immediately formed. A stock ferrofluid of known MNP concentration is then prepared by dispersing the nanoparticles in kerosene using oleic acid as surfactant. In order to do this, 22.5 mL of oleic acid (A16663, Alfa Aesar) is added and the aforementioned solution is stirred vigorously to mix. It is then maintained at near boiling for ~ 2 h until all the ammonia has evaporated. The solution is then cooled to room temperature, 367.5 mL of kerosene (K-1 grade, Sunnyside, Wheeling, IL, USA) is added, and the mixture is stirred vigorously until the kerosene is emulsified. When this emulsion is allowed to settle overnight, the MNPs are exchanged from the water to the kerosene. Thus, the kerosene along with the MNPs moves to the top while transparent water is observed at the bottom. The kerosene-based ferrofluid, thus prepared, is decanted off. Dynamic Light Scattering (Malvern ZetaSizer^TM^ Nano ZS-90, Westborough, MA, USA) shows that the hydrodynamic diameter of the MNPs in this ferrofluid is ~15 nm.

### 3.2. Mold Fabriction

The molds are 15 mm square holes cut in a 3 mm thick polymethyl methacrylate (PMMA) sheet using a laser cutter (Epilog, Golden, CO, USA). The bottoms of the molds are made of plastic cover slips having a thickness of ~250 µm (VWR, Radnor, PA, USA). For samples with heterogeneities in MNP concentration, about half of a coverslip is covered with a rectangular NdFeB permanent magnet (Grade N52, 1/2'' × 1/2'' × 1/8'', gold plated, product # B882G-N52, K&J Magnetics, Pipersville, PA, USA). The magnet produces the gradient in magnetic field strength that is required for magnetophoresis.

### 3.3. Preparation of Nanocomposites

The PDMS pre-deposition solution should have a low viscosity to ensure a thorough dispersion of MNPs in the PDMS. Thus, we begin with a solution containing 1:1 (*w*/*w*) of PDMS and kerosene. In order to accomplish this, the stock ferrofluid is first diluted in kerosene to obtain the desired weight fraction of MNPs to PDMS, *ψ*. The two parts of the PDMS elastomer kit (Sylgard^TM^ 184, Dow Corning, Midland, MI, USA) are separately mixed in the prescribed ratio of 9:1 (*w*/*w*). The PDMS is then dissolved in the diluted ferrofluid so that the final solution has equal weights of kerosene and PDMS. The solution is vigorously stirred until it appears homogeneous under an optical microscope, then transferred into the PMMA molds. The molds are subsequently placed in a vacuum oven and subjected to an absolute pressure of ~5 kPa for 24 h at room temperature. Within the first 30 min, most MNPs in the solution move to the region experiencing the strongest field strength, *i.e.*, close to the edges of the magnet. Within 24 h, all the kerosene is evaporated from the solution (determined by weight measurements). Elevated temperatures are avoided in this phase so that the polymer does not cross-link substantially. This is because cross-linking impedes (1) complete magnetophoresis of all MNPs, and more importantly, (2) the evaporation of kerosene, leading to bubble formation. After the first 24 h, the samples are subjected to a temperature of 60 °C for another 24 h so that the elastomer is fully cured.

### 3.4. Nanoindentation

**Figure 5:**
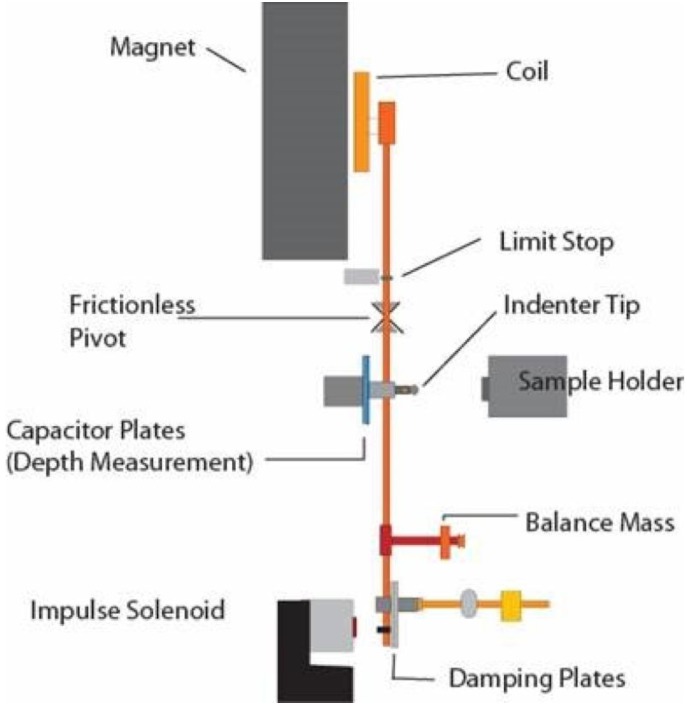
Schematic of the NanoTest^®^ instrument. The indenter is attached to a pendulum that can rotate around a virtually frictionless pivot. The indenter is loaded against the sample by passing a current through the coil, which is then drawn to the permanent magnet. Displacement of the indenter into the sample is measured by the variation in voltage between the capacitor plates.

Instrumented nanoindetation tests are performed using a NanoTest^®^ instrument (Micro Materials Limited, Wrexham, UK). A schematic of the instrument is provided in Figure 5. In this study, an indenter with a spherical diamond tip of 5.0 µm diameter is used to penetrate the sample surface at various points. Each indentation involves loading of the specimen till the indenter penetrates to a depth of 4.0 µm, holding the load constant to observe material creep and, finally, unloading. A loading time of 30 s, unloading time of 10 s and a creep dwell period of 80 s are employed for all indentations. The loading is performed by electromagnetic actuation of a pendulum that bears the indenter. A capacitive transducer yields the penetration depth at a given load. Thus, the data recorded from the experiments provide a load-indentation depth relationship, e.g., the ones presented in Figure 2a. The data are corrected for thermal drifts and the frame compliance of the instrument. Details regarding the operating principles have been provided previously [49]. The load-indentation depth relationship is first used to determine the stiffness *S* from the span of the unloading curve between 20% and 80% of maximum load. Next, the Oliver–Pharr method [50,51] is used to determine the reduced modulus *E*_r_. The latter is directly related to the elastic modulus *E*_s_ of the sample at the point of interest by:

(1) $$\frac{1}{E_{r}}=\frac{1-{\text{ν}}_{s}^{2}}{E_{s}}+\frac{1-{\text{ν}}_{i}^{2}}{E_{i}}$$

where *E_i_* and ν*_i_* denote the elastic modulus and Poisson’s ratio of the indenter and ν_s_ is the Poisson’s ratio of the sample. For diamond, *E_i_* = 1140 GPa and ν*_i_* = 0.07 As our samples are much softer with *E_i_* ≈ 10 MPa, the second term in Equation (1) above is ignored.

We realize that the *E*_r_ data are not a measure of the stiffness at a location on the surface where the indentation is performed. Rather, it is the effective modulus of a thin layer of material close to the surface. Considering that the indentation depth is 4.0 µm and the indenter diameter is 5.0 µm, the thickness of such a layer is smaller than 50 µm [52]. We consider the determined *E*_r_ to be a representative stiffness of the material at the surface.

## 4. Conclusions

Our findings establish the efficacy of using a magnetic field to control the concentration distribution of a cross-linking impeder as a means for patterning the stiffness of elastomers. We demonstrate the concept using the described PDMS-MNP system due to the ability of MNPs to readily impede cross-linking in PDMS. However, the possibility of functionalizing MNPs ensure its adaptability to several other polymer systems. Such magnetic control presents significantly more flexible patterning capabilities than conventional methods for functional grading. Possible patterns extend well beyond the unidirectional gradation allowed by current technology. The use of nanoparticles enables microscale spatial resolution in stiffness to be achieved. Thus, the method can span several length scales ranging from a micron to several millimetres, depending on the geometry of the magnetic field used. The microscale resolution enables incorporation of functional grading in microfabrication techniques. Further, it can significantly enhance current additive manufacturing systems by adding the capability to pattern heterogeneities in stiffness.
